# Divergent performance of vaccines in the UK autumn 2023 COVID booster campaign

**DOI:** 10.1016/S0140-6736(24)00316-7

**Published:** 2024-03-11

**Authors:** Marianne Shawe-Taylor, David Greenwood, Agnieszka Hobbs, Giulia Dowgier, Rebecca Penn, Theo Sanderson, Phoebe Stevenson-Leggett, James Bazire, Ruth Harvey, Crick COVID serology pipeline, Legacy Investigators, Vincenzo Libri, George Kassiotis, Steve Gamblin, Nicola S Lewis, Bryan Williams, Charles Swanton, Sonia Gandhi, Edward J Carr, Mary Y Wu, David LV Bauer, Emma C Wall

**Affiliations:** 1 The Francis Crick Institute, 1 Midland Road, London NW1 1AT; 2 National Institute for Health Research (NIHR) University College London Hospitals (UCLH) Biomedical Research Centre and NIHR UCLH Clinical Research Facility; 3 COVID Surveillance Unit, The Francis Crick Institute, 1 Midland Road, London, NW1 1AT; 4 Worldwide Influenza Centre, The Francis Crick Institute, 1 Midland Road, London, NW1 1AT; 5 Department of Infectious Disease, St Mary's Hospital, Imperial College London, London; 6 University College London, London, UK; 7 Genotype-to-Phenotype 2 Consortium (G2P2-UK)

On 13 December 2023, the World Health Organisation’s Technical Advisory Group on COVID-19 Vaccine Composition (TAG-CO-VAC) reiterated their guidance^1^ from May 2023 recommending updating vaccines to better induce neutralising antibodies against the omicron XBB.1 lineage and its descendants that were in circulation at the time. TAG-CO-VAC suggested the use of monovalent XBB.1 or descendant lineage (e.g. XBB.1.5), and specifically recommended “moving away” from the inclusion of the ancestral “index” virus first detected in 2019 in vaccines.

In countries such as the United States and Japan, bivalent vaccines incorporating older omicron lineages and the index virus were not used for the Autumn 2023 “booster” programmes. In contrast, in the United Kingdom, the Joint Committee on Vaccination and Immunisations (JCVI) adopted a pragmatic approach^2^ and recommended the continued use of stocks of prior bivalent mRNA vaccines in addition to the newer monovalent XBB.1.5-encoding mRNA vaccines, on the basis that both showed efficacy during mid-2023 — despite the prior bivalent vaccines encoding the Spike protein of viruses that last circulated in mid-2022 and early 2020 (Figure 1A).

SARS-CoV-2 has evolved considerably since these decisions were first made: over the course of 2023, the XBB lineage accumulated several mutations in its Spike protein that have been shown to incrementally decrease neutralising capacity of serum from infected and vaccinated individuals^3^. Notably, a new lineage of omicron, BA.2.86, was detected in August 2023: while a descendant of the omicron BA.2 lineage, its sequence is more divergent from XBB than omicron BA.1 was from delta when BA.1 first appeared (Figure 1B). BA.2.86 is now the dominant lineage in Europe as of December 2023 after acquiring a number of adaptive mutations (JN.1 variant). These changes have implications for both vaccine-induced immunity, as well as for monoclonal antibody therapies used for highly vulnerable individuals ^4,5^.

In this Correspondence, we report active surveillance of the Crick/UCLH Legacy cohort (NCT04750356) in the United Kingdom that has permitted near head-to-head comparison of the boosting efficacy of the newer XBB monovalent vaccines and the older bivalent ancestral+BA.4/5 vaccines against a range of Omicron sub-variants that capture the evolutionary diversity of SARS-CoV-2 across 2023 — as well as their effect on mucosal immunity ^6^.

Across our cohort, 71 participants reported receiving a fifth dose COVID-19 “booster” vaccination in September and October 2023 (Table 1, Appendix p2). Remarkably, the administration of the different mRNA vaccine compositions was nearly sequential (Figure 1C): The older bivalent ancestral+BA.4/5 vaccine was administered to 50 participants (50/71; 70%) presenting for vaccination between 15 September 2023 and 12 October 2023, while the newer monovalent XBB vaccine was administered to 21 participants (21/71; 30%) from 10 October 2023. Serum and nasal swabs were collected a median of 4 days prior to 5^th^ dose vaccination for a total of 56 participants (56/71; 79%), and a median of 18 days following vaccination for a total of 68 participants (68/71; 96%). Of these, paired serum samples were collected from 53 participants (Table 1, Appendix p2). Participants were grouped according to their 5^th^ dose vaccine type, and the neutralisation capacity of both serum and mucosal samples collected pre- and post-vaccination was assessed using the Crick’s live virus neutralisation assay, calibrated to the WHO International Standard for anti-SARS-CoV-2 antibody^7,8^. Prior to 5^th^ dose vaccination, both groups equally neutralised all variants tested — unsurprising, given their similar age, infection, and vaccination histories (Table 1, Appendix p2, 3). Despite its recent emergence, all participants sampled (56/56; 100%) had detectable neutralising antibodies against BA.2.86 *prior* to vaccination, with a median IC_50_ of 494 (IQR: 370-1206).

Following fifth dose vaccination, serum from recipients of both vaccines had significantly increased ability to neutralise all variants (Figure 1D, median increase 1.9-fold to 9.1-fold, p≤0.024 for all variants). Analysis of paired samples gave the similar result (p<0.001 for all variants, Supplementary Figure 1, Appendix page 4). However, comparing post-vaccination titres between these groups (Figure 1E) revealed that those who received the newer monovalent XBB.1.5 vaccine had ~3.5-fold higher titres against XBB.1.5 (95%CI: 1.6-6.5) and BQ.1.1 (95%CI: 1.6-7.7). Conversely, those who received the bivalent ancestral+BA.4/5 vaccine did not have an increased ability to neutralise the ancestral virus (p=0.774) or BA.5 (p=0.927) relative to those who received the monovalent XBB.1.5 vaccine, despite those antigens being encoded in the bivalent vaccine they received.

Both vaccines provided a similar, significant increase in titres against the newly-emerged BA.2.86 variant: 3.3-fold (95%CI: 2.1-4.4) for the bivalent ancestral+BA.4/5 vaccine and 3.4-fold (95%CI: 1.6-6) for the monovalent XBB.1.5 vaccine, with titres following vaccination reaching a median IC_50_ of 1401 (IQR: 761-2120) and 2041 (IQR: 1140-2884), respectively, for each vaccine. When compared to serum taken in June-July 2023 from younger participants who were ineligible for the UK’s “booster” vaccination in both 2023 and 2024 (fourth and fifth doses; Supplementary Figure 2, Supplementary Table 1, Appendix p4, 8), boosted participants have significantly higher neutralising antibodies against all variants post-5^th^ dose (p<0.001 in all cases), and even had higher titres against more recent Omicron variants (XBB.1.5 and BA.2.86) than their booster-ineligible counterparts prior to fifth dose vaccination (p≤0.033), suggesting a sustained advantage from having received a fourth dose vaccination in Autumn 2022.

It is important to note that all study participants had detectable neutralising antibodies against BA.2.86 prior to its emergence in August 2023 (Supplementary Figure 2, Appendix p4), including those ineligible for booster vaccinations who were sampled in June-July 2023. Among those who received booster vaccination, absolute neutralisation titres are substantially higher than estimates of correlates of protection for alpha^9^, delta^10^, and models for omicron^11^, suggesting the JCVI aim to protect clinically vulnerable adults from hospitalisations associated with COVID-19 infection, and reduce both nosocomial transmission and sickness absence in healthcare workers and carers through further vaccination this winter is likely to be effective.

Omicron variants are highly transmissible, in part due to their ability to replicate in the upper respiratory tract^12^, where mucosal immunity may play a greater role in preventing (even mild) infection and transmission. We therefore measured neutralising ability of nasopharyngeal samples before and after vaccination (Supplementary Figure 3A, Appendix p5): we found significantly increased neutralisation against the cognate XBB.1.5 virus (p=0.009) as well as the earlier BA.2 (p=0.011) and BA.5 (p=0.044) variants in participants who received the monovalent XBB.1.5 booster, whereas bivalent ancestral+BA.4/5 vaccine recipients did not similarly increase mucosal neutralisation. Importantly, neither group of vaccine recipients significantly increased mucosal neutralisation of the newly-emerged BA.2.86 variant (p=0.175 & p=0.380), though the proportion of mucosal samples with detectable neutralisation activity against BA.2.86 increased when considering all vaccine types together (Supplementary Figure 3B, Appendix p5, McNemar’s test p=0.035).

While these data are consistent with our prior report showing increase in mucosal neutralisation following intramuscular vaccination^12^, the lack of a substantial boost to mucosal neutralisation of BA.2.86 suggests that current vaccines are likely to less effective at preventing asymptomatic or mild illness and onwards transmission. Consequently, the primary benefit of vaccination remains individual protection against severe disease — something that remains elusive for a number of extremely clinically-vulnerable populations^13^, who respond poorly to vaccination.

For these vulnerable populations, there are limited treatment options since many are unable to take oral Paxlovid (nirmeltrivir/ritonavir) due to interactions between ritonavir and their other essential medications. We tested the neutralising efficacy of UK licensed therapeutic monoclonal antibodies against a panel of SARS-CoV-2 variants, including BA.2.86. Similar to our previous findings on BQ.1.1 ^13^, we found neither casirivimab nor imdevimab alone or in combination neutralised BA.2.86 (Supplementary Figure 4A, Appendix p6). However, sotrovimab retained neutralising activity, but at concentrations close to serum concentrations at 30 days post-infusion (Supplementary Figure 4B, Appendix p6). While much has been made about a “loss” of neutralisation vs. BA.2.86, this is on a similar order to what we observed previously against BQ.1.1 (a BA.5 descendant that circulated in autumn 2022 in the UK) when sotrovimab was in use in the UK. In the absence of clinically licensed alternative treatments for this population and the challenges of pragmatic clinical trials amidst a rapidly changing virus variant landscape, we suggest that updates to treatment guidelines should continue to be informed by both *in vitro* and ‘real-world’ data^14^ — while prioritising trials of increased dosages and rapid development and deployment of new drugs.

In summary, the continuing ability of mRNA vaccines to induce broadly neutralising responses to antigenically distinct variants, in the face of rapid viral evolution remains one of the most remarkable phenomena from the COVID-19 pandemic. However, increasingly narrow strain selection for 2024-5 is not without risk, and active surveillance to guide vaccine strain selection remains essential as evolving human immunity to SARS-CoV-2 risks selecting newer, antigenically-distinct variants over the coming year(s).

Our study is observational and our analysis limited to those who have received their recommended booster vaccinations. While possible that some participants were infected asymptomatically with SARS-CoV-2 in the interval between pre- and post-vaccination sampling, we do not find a concomitant boost of mucosal immunity characteristic of infection^6^, suggesting the effects we observe are driven by parenteral vaccination.

For winter 2023-4, the situation in the UK could have been substantially different if either the newly-emerged BA.2.86 were substantially more antigenically distinct, or if descendants of XBB lineages with progressively more immune escape mutations continued to dominate. In the latter case, those who received the better-matched XBB.1.5 boosters may have had an advantage in immunity over those who received older vaccines. Our data suggest an important lesson for future campaigns: in practice, those who presented earlier for 5^th^ dose vaccination received these older vaccines (Figure 1C); in our cohort, 75% of these older vaccines were given to healthcare workers (Table 1, Appendix p2) who were vaccinated earlier, whereas the 68% of the newer vaccines were given to non-healthcare workers at the Crick who were vaccinated later in the community.

Ultimately, variant-proof COVID-19 treatments and strain-agnostic next-generation vaccines that aim to halt transmission through highly-effective mucosal neutralisation will be required. In the absence of such a vaccine, optimisation of vaccine strain selection, seasonal vaccination programme delivery, and critical evaluation of their impacts, should be prioritised.

## Supplementary Material

Supplementary Materials

## Figures and Tables

**Figure 1:**
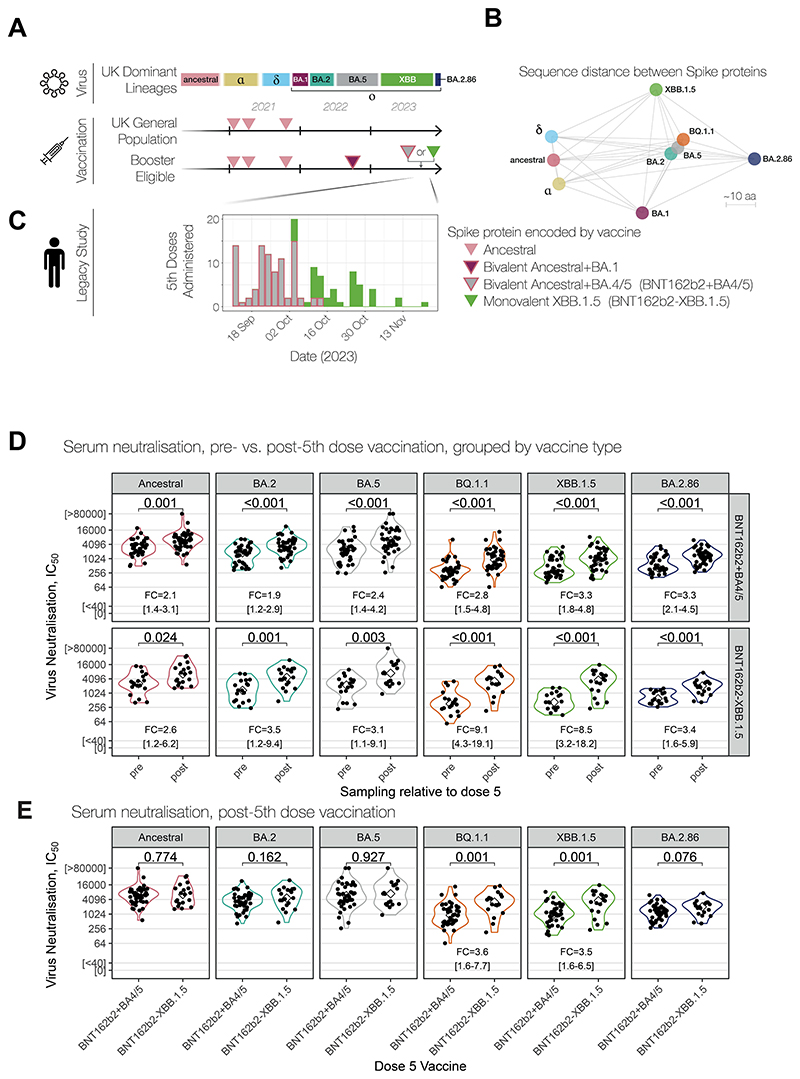
Divergent performance of vaccines in the UK autumn 2023 COVID booster campaign. (**A**) Timeline of circulating SARS-CoV-2 variant lineages and the UK COVID-19 vaccination programme. (**B**) Visualisation of the sequence differences between SARS-CoV-2 variants. Distance scale in amino acids (‘aa’) is indicated, which does not necessarily reflect antigenic distances. (**C**) Fifth doses administered to participants of the UCLH-Crick Legacy study, coloured by vaccine type as indicated. (**D**) Serum neutralisation titres comparing pre- and post-fifth dose, grouped by type of fifth dose vaccine received. (**Eborj**) Serum neutralisation titres following fifth dose vaccination in eligible participants compared between fifth dose vaccine received. In all panels, P-values were determined using unpaired two-tailed Wilcoxon signed-rank tests, and where calculated, the median fold-change (‘FC’) between pre- and post-vaccination titres and the 95% confidence interval (see Methods, Appendix page 10-11) are indicated. An analysis of paired data is shown in Supplementary Figure 1 (Appendix p3).
