# Supplementary material for: Divergent performance of vaccines in the UK autumn 2023 COVID booster campaign
Source: Lancet. Author manuscript; Available in PMC 2024 Nov 18. (PMC7616823; doi:10.1016/S0140-6736(24)00316-7)
Supplement: Supplementary Materials [file EMS200022-supplement-Supplementary_Materials.pdf]

Supplementary appendix for

Divergent performance of vaccines in the UK autumn 2023 COVID booster campaign

Marianne Shawe-Taylor[1,2]*, David Greenwood[1]*, Agnieszka Hobbs[1,2]*, Guilia Dowgier[1,2]*, Rebecca Penn[1], Theo Sanderson[1], Phoebe Stevenson-Leggett[1,2], James Bazire[1,2], Ruth Harvey[1,4], *Crick COVID serology pipeline, Legacy Investigators*, Vincenzo Libri[2], George Kassiotis[1,5], Steve Gamblin[1], Nicola S Lewis[1,3], Bryan Williams[2,6], Charles Swanton[1,6], Sonia Gandhi[1,6], Edward J Carr[1,6]**, Mary Y Wu[1,2]**, David LV Bauer[1,7]**, Emma C Wall[1,2]**

*These authors contributed equally
**These authors contributed equally

1. The Francis Crick Institute, 1 Midland Road, London NW1 1AT
2. National Institute for Health Research (NIHR) University College London Hospitals (UCLH) Biomedical Research Centre and NIHR UCLH Clinical Research Facility,
3. COVID Surveillance Unit, The Francis Crick Institute, 1 Midland Road, London, NW1 1AT
4. Worldwide Influenza Centre, The Francis Crick Institute, 1 Midland Road, London, NW1 1AT
5. Department of Infectious Disease, St Mary's Hospital, Imperial College London, London
6. University College London, London, UK
7. Genotype-to-Phenotype 2 Consortium (G2P2-UK)

Correspondence to david.bauer@crick.ac.uk and emma.wall@crick.ac.uk

Contents:

Table 1

Supplementary Figures 1-4

Supplementary Methods

Supplementary Tables 1-2

Table 1. Description of cohort characteristics grouped by fifth dose vaccine type and those sampled pre- and post-vaccination.

Characteristic	BNT162b2+BA4/5		BNT162b2-XBB.1.5	
	**Paired**, N = 36[1]	**Pre/Post only**, N = 14[1]	**Paired**, N = 17[1]	**Pre/Post only**, N = 4[1]
Sampling relative to dose 5				
Pre	36 (100%)	2 (14%)	17 (100%)	1 (25%)
Post	36 (100%)	12 (86%)	17 (100%)	3 (75%)
Sex				
Female	24 (67%)	12 (86%)	11 (65%)	3 (75%)
Male	12 (33%)	2 (14%)	6 (35%)	1 (25%)
Median age (years) [IQR]	55 [45-61]	51 [45-56]	55 [45-60]	48 [33-61]
Site				
CNWL[2]	8 (22%)	3 (21%)	2 (12%)	1 (25%)
Crick[3]	12 (33%)	2 (14%)	10 (59%)	2 (50%)
Ealing & NWP[4]	0 (0%)	0 (0%)	0 (0%)	0 (0%)
UCLH[5]	16 (44%)	9 (64%)	5 (29%)	1 (25%)
anti-N IgG at latest visit				
negative	3 (8.3%)	1 (7.1%)	0 (0%)	1 (25%)
positive	33 (92%)	13 (93%)	17 (100%)	3 (75%)
N. episodes of infection	2.00 [1.00-3.00]	2.00 [1.25-2.00]	2.00 [1.00-2.00]	3.00 [2.50-3.00]
Smoking status				
Never Smoker	26 (72%)	11 (79%)	11 (65%)	4 (100%)
Ex-smoker	9 (25%)	3 (21%)	5 (29%)	0 (0%)
Current smoker	1 (2.8%)	0 (0%)	1 (5.9%)	0 (0%)
Comorbidity (any)	21 (58%)	6 (43%)	11 (65%)	2 (50%)
Type 1 Diabetes	0 (0%)	0 (0%)	0 (0%)	0 (0%)
Type 2 Diabetes	0 (0%)	0 (0%)	0 (0%)	0 (0%)
Cancer/Stroke/Heart problems	4 (11%)	1 (7.1%)	2 (12%)	0 (0%)
High blood pressure	7 (19%)	1 (7.1%)	4 (24%)	0 (0%)
Asthma/COPD	7 (19%)	3 (21%)	0 (0%)	1 (25%)

[1]n (%); Median [25%-75%]; [2]*Camden and Northwest London Healthcare trust (HCW);* [3] *Francis Crick Institute (predominately non-HCW);* [4]*Ealing and Northwick Park hospitals (HCW);* [5]*University College London Hospitals (HCW)*

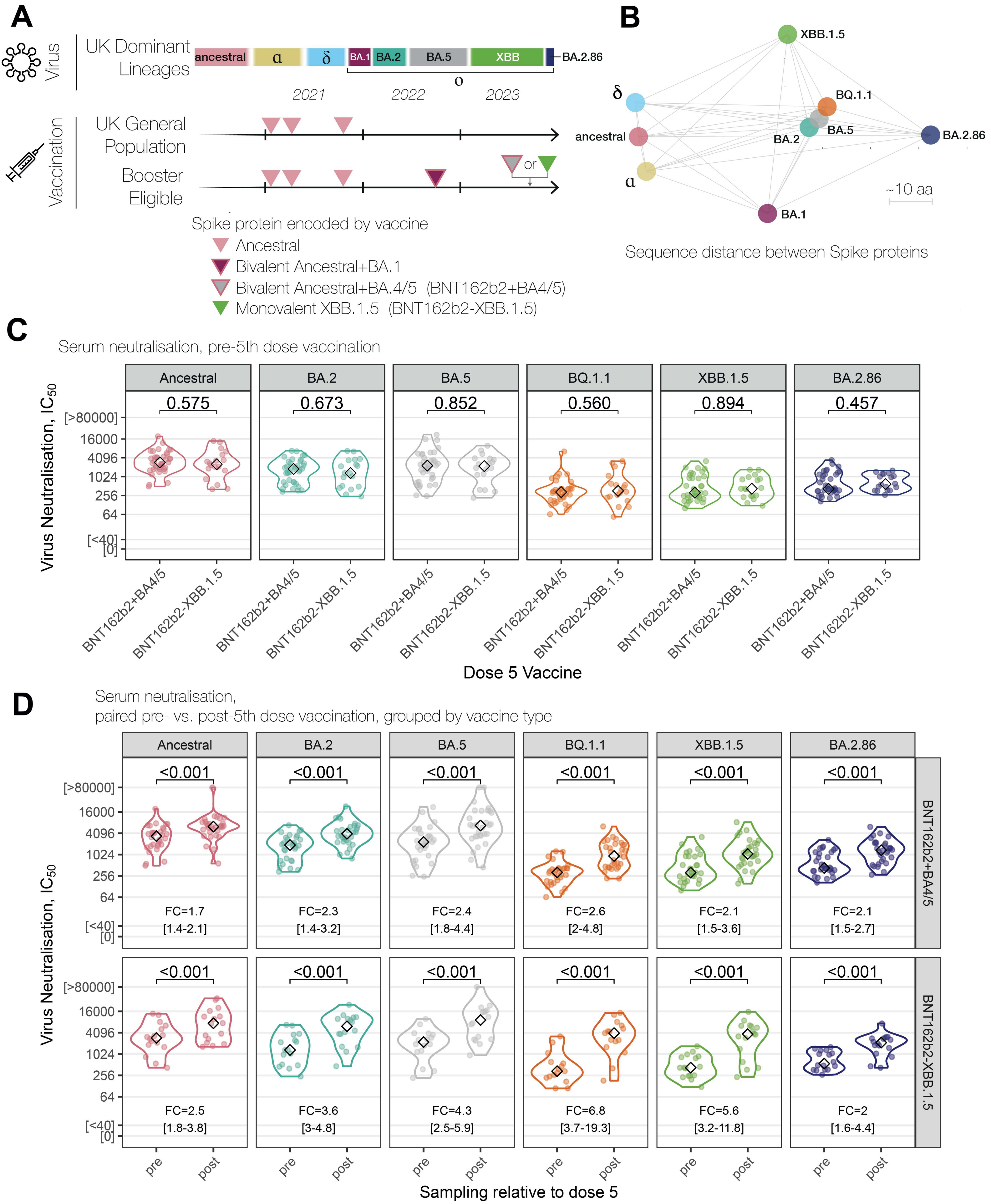

Supplementary Figure 1. (**A**) Timeline of circulating SARS-CoV-2 variant lineages and the UK COVID-19 vaccination programme. (**B**) Visualisation of the sequence differences between SARS-CoV-2 variants. Distance scale in amino acids (‘aa’) is indicated, which does not necessarily reflect antigenic distances. (**C**) Serum neutralisation in pre-5th dose samples, comparing the baseline in participants subsequently administered either the BA.5 bivalent or XBB.1.5 monovalent vaccine. (**D**) Serum neutralisation titres in a subset of 46 participants comparing paired pre- and post-fifth dose, grouped by type of fifth dose vaccine received, using a paired two-tailed Wilcoxon signed rank test.

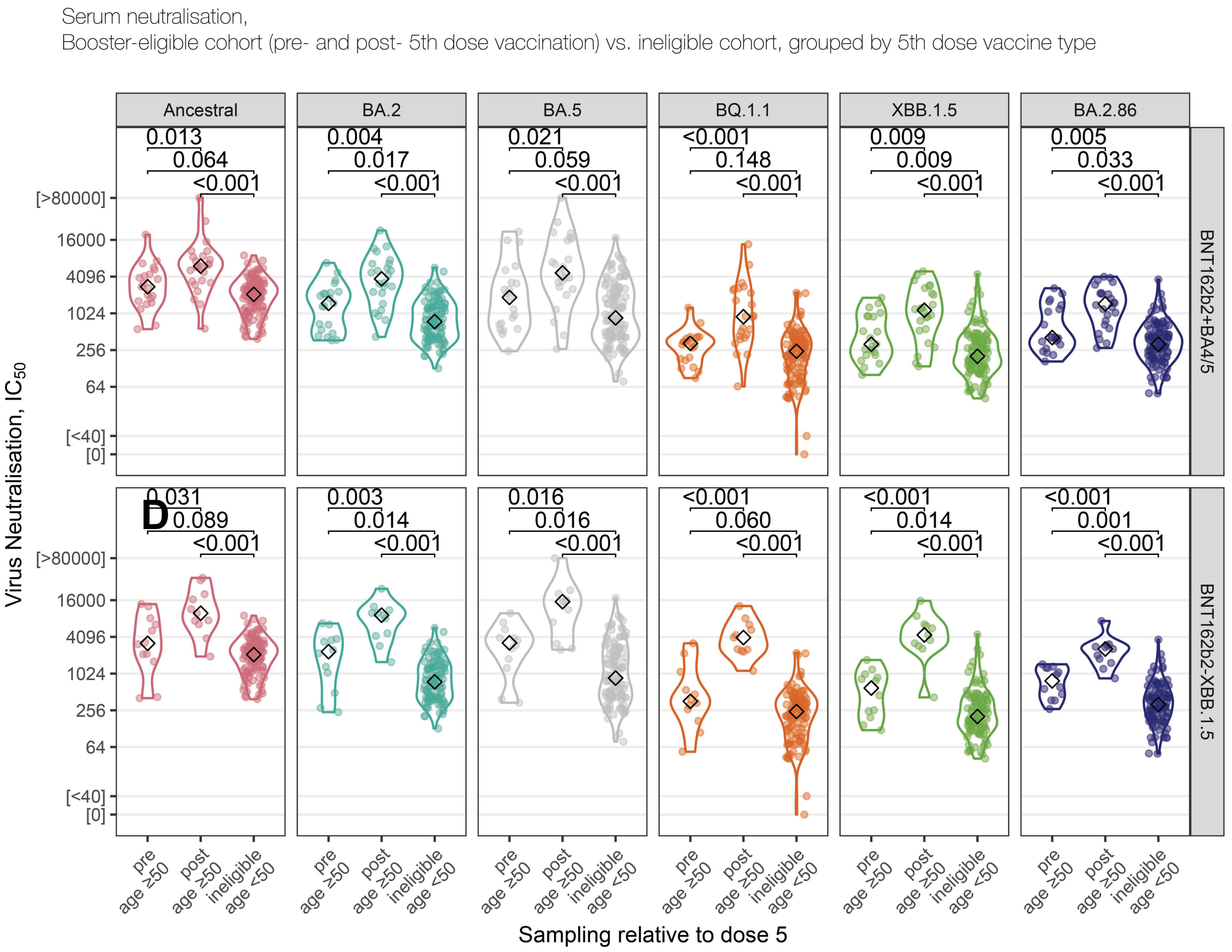

Supplementary Figure 2. Serum neutralising antibody titres are significantly higher in recipients of COVID boosters compared to those ineligible for booster vaccination. Neutralising antibody titres are shown for the booster-eligible cohort (pre- vs. post-fifth dose vaccination) vs. the booster-ineligible cohort, grouped by fifth dose vaccine type (row). P-values were adjusted for multiple testing using a Holm-Bonferroni correction.

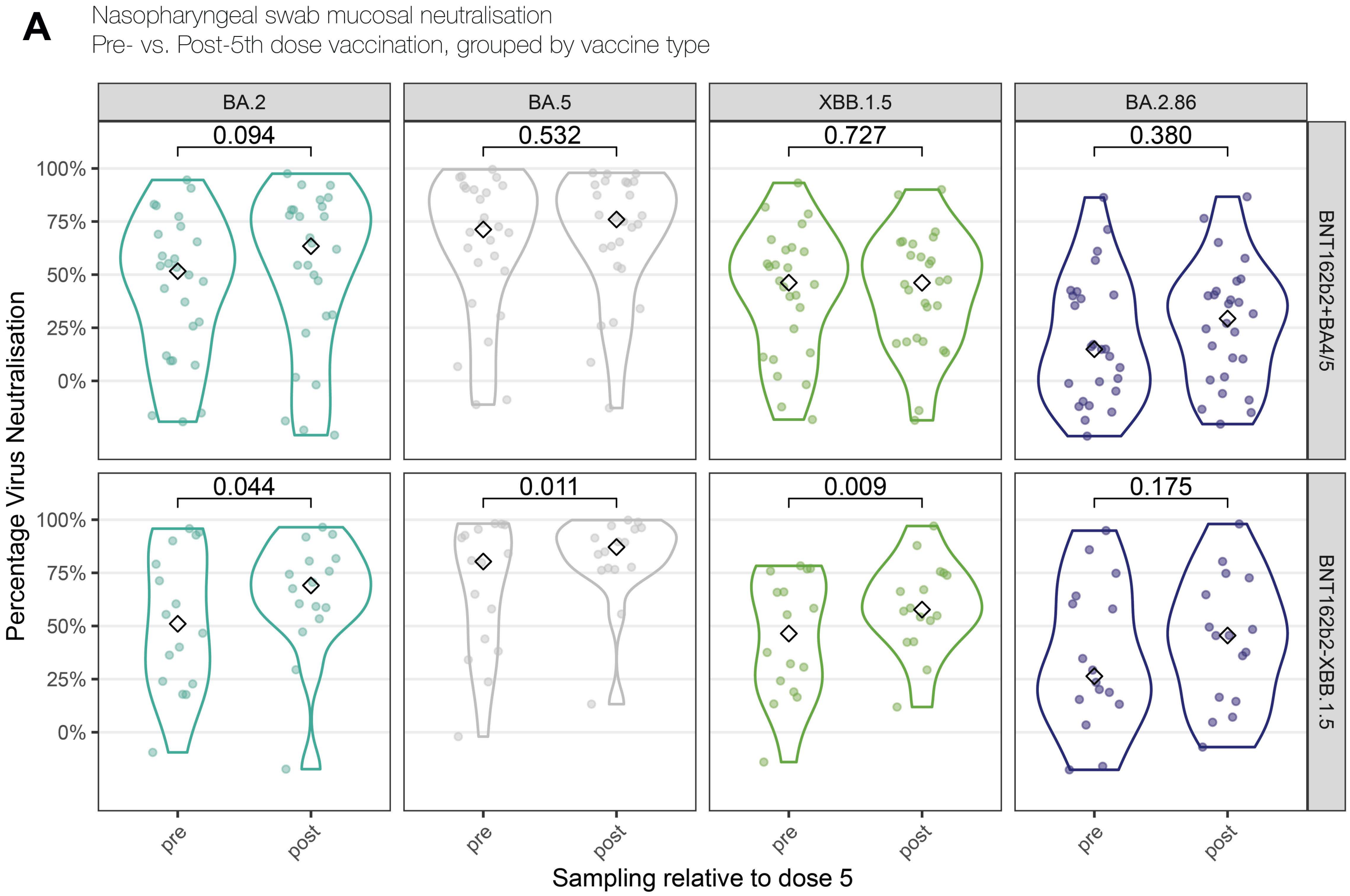

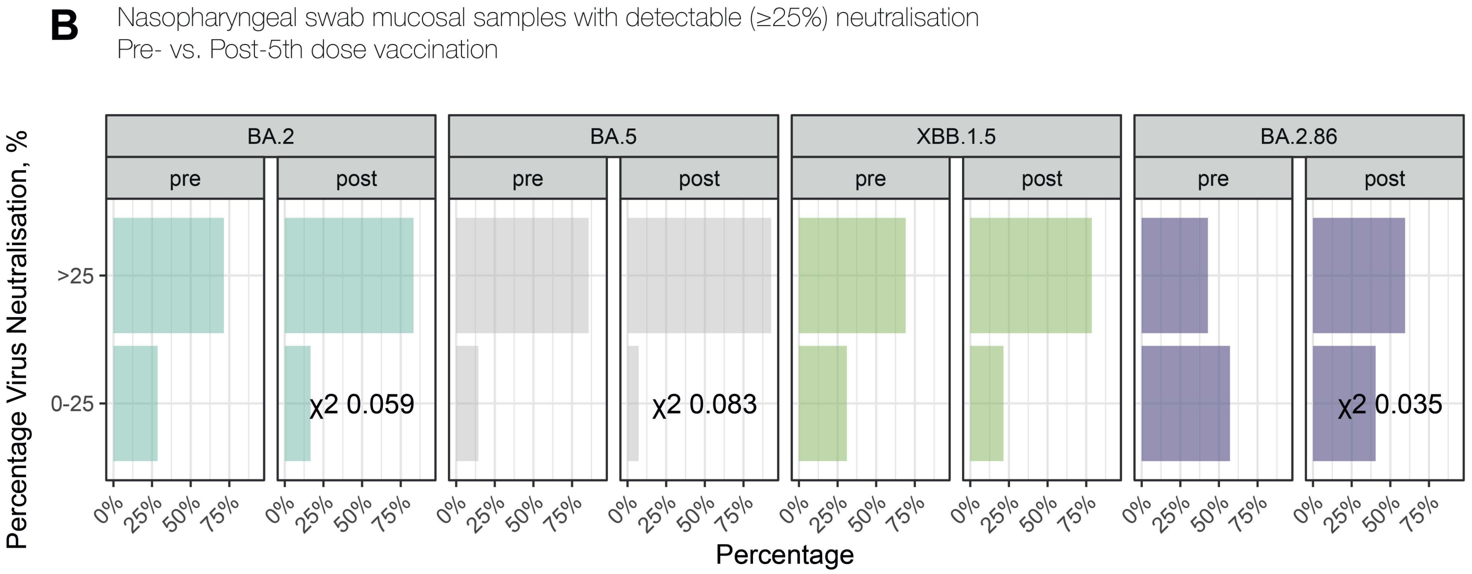

Supplementary Figure 3. Mucosal neutralising antibody titres against XBB.1.5 are boosted in recipients of the monovalent XBB.1.5 vaccine. (**A**) Neutralising antibody titres of nasopharyngeal swab samples from participants pre- and post-fifth dose vaccination, grouped by vaccine type. P-values were determined using a unpaired two-tailed Wilcoxon signed-rank tests. (**B**) Number of participants with detectable (>25%) neutralisation of SARS-CoV-2 variants compared pre- and post-fifth dose vaccination, irrespective of vaccine type. P-values from McNemar's test of paired categorical data are indicated (χ^2).

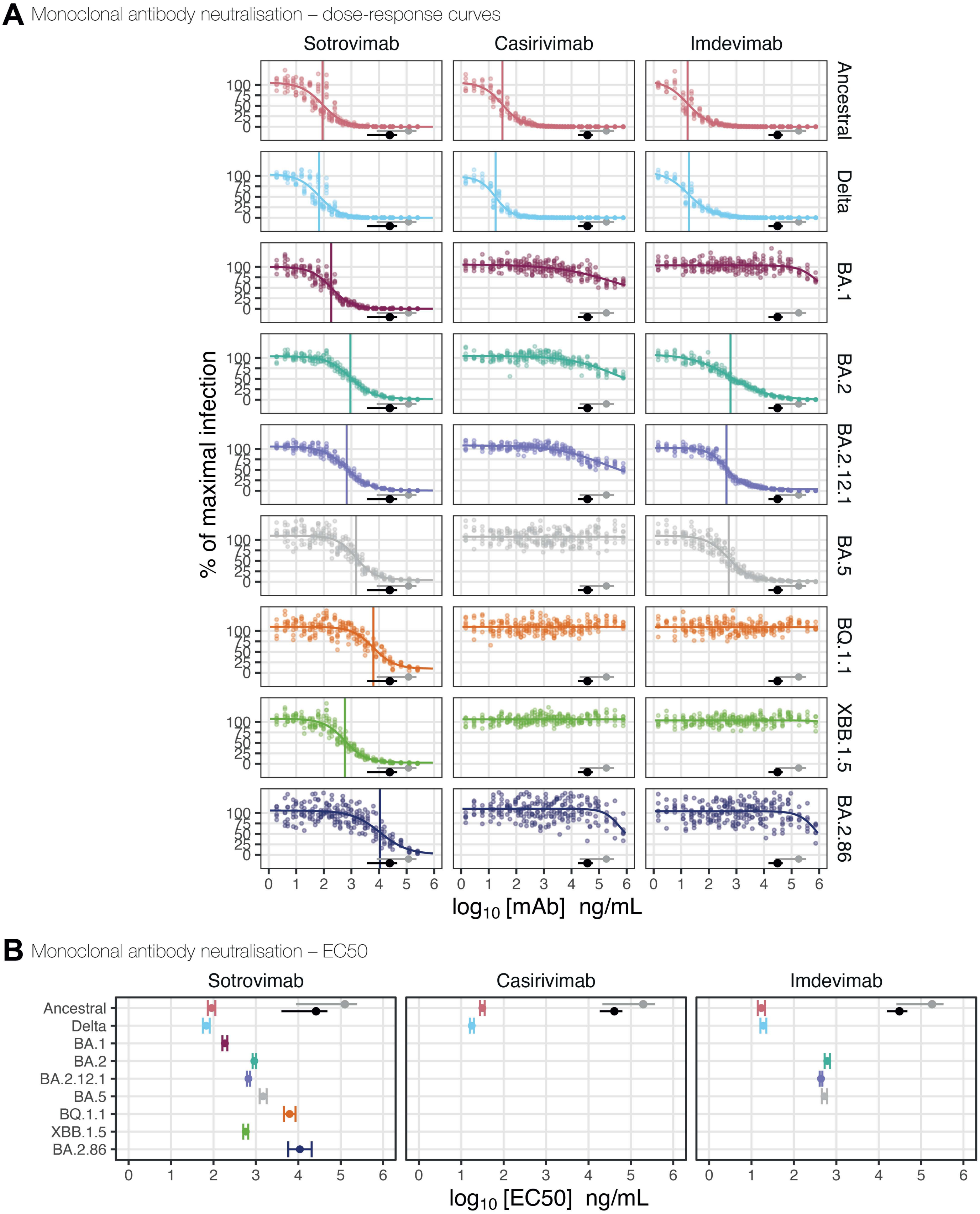

Supplementary Figure 4. Therapeutic monoclonal antibody neutralisation of SARS-CoV-2 variants. (**A**) Dose-response curves fit to monoclonal antibody neutralisation of SARS-CoV-2. Vertical lines indicate the EC_{50} of the monoclonal antibody against the indicated variant. (**B**) Summary plot of EC_{50} values for each monoclonal. In both panels, pharmacokinetic data representing the mean serum concentration 1 day post-infusion (grey) and 28 days post-infusion (black) are overlaid on each plot.

Methods

Clinical cohort

The Legacy study (NCT04750356) is a prospective observational cohort, established in January 2021. Extensive descriptions of the cohort can be found in our prior interim reports[2,3,6–8]. In the UK, from September 2023, healthcare workers, adults over 65 years, and those with either immunocompromise or caring responsibilities were offered a dose of COVID-19 vaccine. The majority of Legacy study participants eligible for this campaign received either a dose of bivalent COVID-19 vaccine containing mRNA encoding Ancestral and Omicron BA.4/5 Spikes (BNT162b2+BA4/5) or a monovalent COVID-19 vaccine containing mRNA encoding the XBB.1.5 Spike (BNT162b2-XBB.1.5) as a fifth dose. Participants were invited for paired pre- and post-vaccination visits approximately 1 week before and 3 weeks after the dose. If an individual was unable to attend pre-vaccination, their dose was not delayed. At each study visit, individuals performed a nasopharyngeal swab into virus transport medium (VTM; MWE Sigma-Virucult), gave details on any recent infection episodes, and had blood drawn for serum for live-virus microneutralisation assays and anti-N IgG detection.

Legacy participants were included in this study if they received their fifth dose of COVID-19 vaccine (BNT162b2+BA4/5 or BNT162b2-XBB.1.5) after August 1st 2023 and had a pre-boost sample taken more than 2 weeks after a previous dose and/or a post-boost sample within 4 weeks of a fifth dose (**Table 1**). We also analysed a subset of participants who contributed paired pre- and post-vaccination serum samples (**Table 1**).

For comparison to the general population, a group of participants were selected who did not receive a fifth or fourth dose of COVID-19 vaccine, were under the age of 50, and had not reported comorbidities that may qualify them for the campaign. To be included, these participants had to have samples available within the date range of the pre-boost samples. We refer to these participants as the “Ineligible” group (**Supplementary Table 1**).

**Characteristic**	**Ineligible**, N = 111[1]
Sex	
Female	85 (77%)
Male	26 (23%)
Median age (years) [IQR]	35 [29-42]
Site	
CNWL[2]	3 (2.7%)
Crick[3]	100 (90%)
Ealing & NWP[4]	2 (1.8%)
UCLH[5]	6 (5.4%)
anti-N IgG at latest visit	
negative	6 (5.4%)
no sample tested	3 (2.7%)
positive	102 (92%)
N. episodes of infection	2.00 [1.00-2.00]
Smoking status	
Never Smoker	85 (77%)
Ex-smoker	20 (18%)
Current smoker	6 (5.4%)
Comorbidity (any)	0 (0%)
Type 1 Diabetes	0 (0%)
Type 2 Diabetes	0 (0%)
Cancer/Stroke/Heart problems	0 (0%)
High blood pressure	0 (0%)
Asthma/COPD	7 (6.3%)

[1]n (%); Median [25%-75%]; [2]*Camden and Northwest London Healthcare trust (HCW);* [3] *Francis Crick Institute (predominately non-HCW);* [4]*Ealing and Northwick Park hospitals (HCW);* [5]*University College London Hospitals (HCW)*

Supplementary Table 1. Description of cohort characteristics for the "booster-ineligible" group sampled June-July 2023.

Virus variants and culture

The live virus isolates used were the same as previously described, and our viral culture technique is unchanged[1,2,7,9,10]. Details of all isolates used in this study, with their Spike mutations are detailed in **Supplementary Table 2** below. Omicron sub-variants isolated at the Francis Crick Institute were collected from Legacy participants reporting acute symptomatic infection, following previously described active surveillance protocols[6].

Strain	Spike Protein Mutations	Isolate Source
Ancestral [hCoV19/England /02/2020]	(none)	Public Health England
Omicron BA.2 [hCoV/England/F CI-179/2022]	T19I, Δ24-26, A27S, G142D, V213G, G339D, S371F, S373P, S375F, T376A, D405N, R408S, K417N, N440K, S477N, T478K, E484A, Q493R, Q498R, N501Y, Y505H, D614G, H655Y, N679K, P681H, N764K, D796Y, Q954H, and N969K	Francis Crick Institute
Omicron BA.5	T19I, Δ24- 26, A27S, Δ69-70, G142D, V213G, G339D, S371F, S373P, S375F, T376A, D405N, R408S, K417N, N440K, L452R, S477N, T478K, E484A, F486V, Q498R, N501Y, Y505H, D614G, H655Y, N679K, P681H, N764K, D796Y, Q954H, N969K	Prof. Alex Sigal, Prof. Tulio de Oliveira, Africa Health Research Institute, Durban, South Africa via the Genotype-to-Phenotype National Virology Consortium (G2P-UK)
Omicron BQ.1.1 [hCoV/England/F CI-190/2022]	T19I, L24-, P25-, P26-, A27S, H69-, V70-, V213G, G339D, R346T, S371F, S373P, S375F, T376A, D405N, R408S, K417N, N440K, K444T, L452R, N460K, S477N, T478K, E484A, F486V, Q498R, N501Y, Y505H, D614G, H655Y, N679K, P681H, N764K, D796Y, Q954H, N969K	Francis Crick Institute
Omicron XBB.1.5	T19I, L24S, P25-, P26-, A27-, V83A, G142D, Y144-, H146Q, Q183E, V213E, G252V, G339H, R346T, L368I, S371F, S373P, S375F, T376A, D405N, R408S, K417N, N440K, V445P, G446S, N460K, S477N, T478K, E484A, F486P, F490S, Q498R, N501Y, Y505H, D614G, H655Y, N679K, P681H, N764K, D796Y, Q954H, N969K	Prof. Gavin Screaton, University of Oxford, Oxford, UK via the Genotype-to-Phenotype National Virology Consortium (G2P-UK)
Omicron BA.2.86 [hCov/England/F CI-200/2023	T19I, R21T, L24S, P25-, P26-, A27-, S50L, H69-, V70-, V127F, G142D, Y144-, F157S, R158G, N211I, L212-, V213G, L216F, H245N, A264D, I332V, G339H, K356T, S371F, S373P, S375F, T376A, R403K, D405N, R408S, K417N, N440K, V445H, G446S, N450D, L452W, N460K, S477N, T478K, N481K, E484K, F486P, Q498R, N501Y, Y505H, E554K, A570V, D614G, P621S, H655Y, N679K, P681R, N764K, D796Y, S939F, Q954H, N969K, P1143L	Francis Crick Institute

Supplementary Table 2. Summary of SARS-CoV-2 variants used, their Spike mutational profile and isolate source.

Visualisation of Spike protein distances

To avoid the inability of a phylogenetic tree to accurately represent recombinant lineages (e.g. XBB), we represented Spike protein distances as a network: pairwise edit distances between Spike amino acid sequences of lineages defined in Nextclade were calculated and

represented in two dimensions using stress-minimization in the SARS CoV-2 Variant Mutation Network tool: https://lineagenetwork.streamlit.app

Anti-nucleocapsid IgG detection

Anti-nucleocapsid IgG (anti-N IgG) detection Anti-nucleocapsid IgG was measured using the Elecsys Anti-SARS-COV-2 assay (Roche; 09203095190) run on a Cobas e411 analyser (Roche) in accordance with the manufacturer's instructions. Serum was used for this immunoassay and results reported as reactive (positive) or non-reactive (negative).

High-throughput live-virus microneutralisation assay for serum and nasopharyngeal samples

High-throughput live-virus microneutralisation assays for serum samples were performed as previously described[1,2,7,9] and the assay for nasopharyngeal samples is reported in [10].

Data analysis, statistics, and availability

Data used in this study were collected and managed using REDCap electronic data capture tools hosted at University College London[11,12]. Data were imported to R from REDCap prior to analysis as previously described[1,7,8]. Data were manipulated, analysed and visualised using *tidyverse* R packages[13] including *dplyr* and *ggplot2*[14,15]. Summary descriptions of the cohort were generated using *gtsummary*[16]. Continuous data were reported as the median value and interquartile range (IQR) or the first and third quartiles (Q1; Q3). Statistical tests were conducted with the *rstatix* R package[17].

Analysis of neutralising antibody titres in serum was performed as previously described without alterations using unpaired two-tailed Wilcoxon signed-rank tests[1,2,7,18,19]. Fold changes (FC) were estimated between groups with a 95% confidence interval (CI) with the *boot* R package using 5000 bootstrap resamples[21].

Mucosal neutralisation capacity was pre-processed as follows: Cell viability for each well was confirmed using a 70% threshold of the plate median of each well's summed cell area (across the 4 replicates). If 2 or more replicates had a cell area below this threshold, the well was

deemed to have failed. Neutralisation percentage, as (100 – Infection), was reported as the median across the four replicates.

To compare mucosal neutralisation capacity between groups, two-tailed Wilcoxon matched pairs signed-rank tests were applied[19,22]. Mucosal neutralisation capacity was also dichotomised into un-detectable (0-25%) and detectable (>25%) neutralisation and McNemar's Chi-squared (χ2) tests for paired categorical data were applied[19,23].

Ethics

The Legacy study was approved by London Camden and Kings Cross Health Research Authority (HRA) Research and Ethics committee (REC, reference 20/HRA/4717) IRAS number 286469 and 156 sponsored by University College London.

Role of the funding source

This work was undertaken at UCLH/UCL who received a proportion of funding from the National Institute for Health Research (NIHR) University College London Hospitals Department of Health's NIHR Biomedical Research Centre (BRC). EW, VL and BW are supported by the Centre's funding scheme. This work was supported jointly by the BRC and core funding from the Francis Crick Institute, which receives its funding from Cancer Research UK, the UK Medical Research Council, and the Wellcome Trust. EJC is supported by an MRC clinician scientist fellowship. DLVB is additionally supported by the Genotype-to-Phenotype National Virology Consortium (G2P-UK), Genotype-to-Phenotype 2 (G2P2-UK) and via UK Research and Innovation and the UK Medical Research Council. This research was funded in whole, or in part, by the Wellcome Trust [CC2166, CC1283, CC1114, CC2230, CC2060, CC2041]. For the purpose of Open Access, the author has applied a CC BY public copyright licence to any Author Accepted Manuscript version arising from this submission. This work was supported by the National Institute for Health Research University College London Hospitals Department of Health's NIHR Biomedical Research Centre (BRC), as well as by the UK Research and Innovation and the UK Medical Research Council (MR/W005611/1, MR/Y004205/1, and MR/X006751/1 to EJC), and by the Francis Crick Institute which receives

its core funding from Cancer Research UK (CC2166, CC1283, CC1114, CC2230, CC2060, CC2041), the UK Medical Research Council (CC2166, CC1283, CC1114, CC2230, CC2060, CC2041), and the Wellcome Trust (CC2166, CC1283, CC1114, CC2230, CC2060, CC2041). The funders of the study had no role in study design, data collection, data analysis, data interpretation, or writing of the report.

All authors had access to the data in the study. The corresponding author and senior authors had final responsibility for the decision to submit for publication.

Consortium Authors

Crick serology pipeline Investigators**:** Ashley S Fowler, Murad Miah, Callie Smith, Mauro Miranda, Philip Bawumia, Harriet V Mears, Lorin Adams, Emine Hatipoglu, Nicola O'Reilly, Scott Warchal, Karen Ambrose, Amy Strange, Gavin Kelly, Svend Kjaer

Legacy Investigators: Rupert CL. Beale, Padmasayee Papineni, Tumena Corrah, Richard Gilson

Author contributions

MS-T - Investigation, project administration, Data Curation, Formal Analysis, Investigation, review & editing

DG - Investigation, Data Curation, Formal analysis, Investigation, review and editing

GD - Investigation, Data Curation

AH - Investigation, Data Curation

RP - Investigation, Data Curation

TS - Investigation, Data Curation

PS-L - Investigation, Data Curation

JB - Investigation, Data Curation

RH - Investigation, Methodology, Resources, Writing – review & editing, Conceptualization

Crick Serology Pipeline Investigators -Investigation, Data Curation, Project administration, Conceptualization, Software, Methodology, Formal Analysis, Validation

Legacy Investigators - Resources, Investigation Project administration, Data curation, Review & editing

VL- Resources, Project administration, Writing – review & editing

GK - Formal Analysis, Writing – review & editing

SGam - Funding acquisition, Project administration, Supervision

NSL - Resources, Project administration, review & editing

BW - Funding acquisition, Project administration, Writing – review & editing, Conceptualization

CSw- Supervision, Funding acquisition, Project administration, Writing – review & editing, Conceptualization. Has access to & has verified underlying data.

SGan - Supervision, Funding acquisition, Methodology, Project administration, Writing – review & editing.

EJC - Data Curation, Writing - original draft. Has access to & has verified underlying data.

MW - Investigation, Methodology, Resources, Writing – review & editing, Conceptualization. Has access to & has verified underlying data.

DVLB - Methodology, Formal Analysis, Visualization, Writing - original draft, review and editing, Conceptualization. Has access to & has verified underlying data.

ECW – Methodology, Conceptualisation, Investigation, Data Curation, Writing - review and editing. Has access to & has verified underlying data.

Supplementary References

1 Carr EJ, Wu MY, Gahir J, *et al.* Neutralising immunity to omicron sublineages BQ.1.1, XBB, and XBB.1.5 in healthy adults is boosted by bivalent BA.1-containing mRNA vaccination and previous Omicron infection. *The Lancet Infectious Diseases* 2023; **23**: 781–4.

2 Wu M, Wall EC, Carr EJ, *et al.* Three-dose vaccination elicits neutralising antibodies against omicron. *Lancet (London, England)* 2022; **399**: 715–7.

3 Carr EJ, Dowgier G, Greenwood D, *et al.* SARS-CoV-2 mucosal neutralising immunity after vaccination. *The Lancet Infectious Diseases* 2023; **0**. DOI:10.1016/S1473-3099(23)00705-3.

4 WHO/BS.2020.2403 Establishment of the WHO International Standard and Reference Panel for anti-SARS-CoV-2 antibody. https://www.who.int/publications/m/item/WHO-BS-2020.2403.

5 WHO/BS/2022.2427: Establishment of the 2nd WHO International Standard for anti-SARS-CoV-2 immunoglobulin and Reference Panel for antibodies to SARS-CoV-2 variants of concern. https://www.who.int/publications/m/item/who-bs-2022.2427.

6 Townsley H, Gahir J, Russell TW, *et al.* COVID-19 in non-hospitalised adults caused by either SARS-CoV-2 sub-variants Omicron BA.1, BA.2, BA.5 or Delta associates with similar illness duration, symptom severity and viral kinetics, irrespective of vaccination history. DOI:10.1101/2022.07.07.22277367.

7 Wall EC, Wu M, Harvey R, *et al.* AZD1222-induced neutralising antibody activity against SARS-CoV-2 Delta VOC. *Lancet (London, England)* 2021; **398**: 207–9.

8 Wall EC, Wu M, Harvey R, *et al.* Neutralising antibody activity against SARS-CoV-2 VOCs B.1.617.2 and B.1.351 by BNT162b2 vaccination. *Lancet (London, England)* 2021; **397**: 2331–3.

9 Wall EC, Wu M, Harvey R, *et al.* Neutralising antibody activity against SARS-CoV-2 VOCs B.1.617.2 and B.1.351 by BNT162b2 vaccination. *Lancet (London, England)* 2021; **397**: 2331–3.

10 Carr EJ, Dowgier G, Greenwood D, *et al.* SARS-CoV-2 mucosal neutralising immunity after vaccination. *The Lancet Infectious Diseases* 2023; **0**. DOI:10.1016/S1473-3099(23)00705-3.

11 Harris PA, Taylor R, Minor BL, *et al.* The REDCap consortium: Building an international community of software platform partners. *Journal of Biomedical Informatics* 2019; **95**: 103208.

12 Harris PA, Taylor R, Thielke R, Payne J, Gonzalez N, Conde JG. Research electronic data capture (REDCap)–a metadata-driven methodology and workflow process for providing translational research informatics support. *Journal of Biomedical Informatics* 2009; **42**: 377–81.

13 Wickham H, Averick M, Bryan J, *et al.* Welcome to the tidyverse. 2019; **4**: 1686.

14 Wickham H, François R, Henry L, Müller K, Vaughan D. Dplyr: A grammar of data manipulation. 2023. https://CRAN.R-project.org/package=dplyr.

15 Wickham H. ggplot2: Elegant graphics for data analysis. 2016. https://ggplot2.tidyverse.org.

16 Sjoberg DD, Whiting K, Curry M, Lavery JA, Larmarange J. Reproducible summary tables with the gtsummary package. 2021; **13**: 570–80.

17 Kassambara A. Rstatix: Pipe-friendly framework for basic statistical tests. 2023. https://CRAN.R-project.org/package=rstatix.

18 Wall EC, Wu M, Harvey R, *et al.* Neutralising antibody activity against SARS-CoV-2 VOCs B.1.617.2 and B.1.351 by BNT162b2 vaccination. *Lancet (London, England)* 2021; **397**: 2331–3.

19 Wilcoxon F. Individual comparisons by ranking methods. *Biometrics Bulletin* 1945; **1**: 80–3.

20 Canty A, Ripley BD. Boot: Bootstrap r (s-plus) functions. 2021.

21 DiCiccio TJ, Efron B. Bootstrap confidence intervals. *Statistical Science* 1996; **11**: 189–212.

22 Carr EJ, Dowgier G, Greenwood D, *et al.* SARS-CoV-2 mucosal neutralising immunity after vaccination. *The Lancet Infectious Diseases* 2023; **0**. DOI:10.1016/S1473-3099(23)00705-3.

23 McNEMAR Q. Note on the sampling error of the difference between correlated proportions or percentages. *Psychometrika* 1947; **12**: 153–7.
